# Experimental Study of the Influence of the Surface Preparation on the Fatigue Behavior of Polyamide Single Lap Joints

**DOI:** 10.3390/ma14041008

**Published:** 2021-02-20

**Authors:** Francesco Musiari, Fabrizio Moroni

**Affiliations:** Department of Engineering and Architecture, University of Parma, Parco Area delle Scienze, 181/A, 43124 Parma, Italy; francesco.musiari@unipr.it

**Keywords:** fatigue, plasma treatment, polyamide

## Abstract

The low quality of adhesion performance on polymeric surfaces has forced the development of specific pretreatments able to toughen the interface between substrate and adhesive. Among these methods, atmospheric pressure plasma treatment (APPT) appears particularly suitable for its environmental compatibility and its effectiveness in altering the chemical state of the surface. In this work, an experimental study on adhesively bonded joints realized using polyamide as substrates and polyurethane as the structural adhesive was carried out with the intent to characterize their fatigue behavior, which represents a key issue of such joints during their working life. The single lap joint (SLJ) geometry was chosen and several surface pretreatments were compared with each other: degreasing, abrasion (alone and followed by APPT) and finally APPT. The results show that the abrasion combined with APPT presents the most promising behavior, which appears consistent with the higher percentage of life spent for crack propagation found by means of DIC on this class of joints with respect to the others. APPT alone confers a good fatigue resistance with respect to the simple abrasion, especially at a low number of cycles to failure.

## 1. Introduction

The growing needs by industry to reduce the weight of structures lead to an effort in using polymeric materials for structural purposes, in applications requesting a low load-carrying capability, and in extending to them the connection strategies mostly reserved to common metallic materials. Among these ones, adhesive bonding plays a significant role in reducing the time for assembly and goes further towards a weight saving of the structures. The main issues involving this connection technique, especially on polymeric adherends, are represented by (i) the low wettability of the surfaces; (ii) the difficulty in adequately estimating the mechanical behavior of the joints under the real work conditions, which they usually undergo. The first one is typically faced through the application of pretreatments on the surfaces aimed to be bonded. With particular reference to polymeric adherends, several pretreatments appeared to be particularly appropriate: oxidation by means of flame treatment, metal–ion treatment, electric corona discharge, application of primers. An eco-friendly method, which was found to be an effective way to increase the surface free energy of polymeric surfaces in order to improve their usability as substrates for adhesively bonded joints, is the atmospheric pressure plasma treatment (APPT). In this work, APPT was applied over substrates realized in polyamide, which was selected as substrate material due to its advantageous mechanical properties/cost ratio. Many works were dedicated to the modifications induced by APPT over thin films [1] or fibers [2]. Gao et al. [3] tested the effect of He/CF_4_ plasma treatment time on the etching rate of PA6 films, finding that it continuously decreased when the treatment time progressively rose from 30 to 90 ms, which was consistent with the trends exhibited by both the surface free energy and the T-peel strength between the films and an adhesive tape. In another work by the same authors [4], an attempt to explore the response of the etching rate by modifying some APPT process parameters (e.g., output power, oxygen flow rate, surface-to-nozzle distance, moisture regain) resulted in an increase in the etching rate with the treatment time until it reached a threshold, while the T-peel strength was found to keep on increasing with the treatment time. The response of both O_2_/N_2_ and NH_3_ microwave plasma treatment over the mechanical response of joints produced bonding substrates realized both in PA12 and in PA11, respectively, using an epoxy adhesive was investigated by Lennon et al. [5]. They found that no changes in surface roughness occurred due to the treatment, while the surface free energy was found to increase with both the power and the treatment time, leading to the conclusion that surface modifications were mostly chemical and not morphological. This was confirmed, with different arguments, also by Hnilica et al., which in [6] documented a marked difference in the surface roughness of PA12 surfaces, achievable by pretreating them with microwave plasma employing Ar/O_2_ or Ar/N_2_, respectively, as process mixtures of gasses, even if the water contact angles remained very similar for both the pretreatments. The main reason for the increase of the wettability recorded when applying a plasma treatment over polyamide surfaces was indeed related to the formation of carboxylic and hydroxylic groups on the surface and, therefore, to an increase of the oxygen content [7]. Mandolfino et al. [8] carried out an optimization of some cold plasma treatment process parameters with respect to the wettability of the surfaces and to the shear strength of some joints produced bonding some substrates realized with both PA6 and PA6.6., respectively, with an acrylic adhesive. By comparing the results of plasma-treated joints and simply abraded specimens, it was possible to conclude how the increased surface roughness produced by the abrasion did not lead to a corresponding increase of strength because of the poor wettability, which inhibited the adhesive from completely fill in the valleys induced over the surface. Nevertheless, the enhancement of the mechanical strength was however not only due to the increase of the wettability, but it was related to an increase of the concentration of C-OH, C = O and O-C = O groups on the surfaces. With regard to the second aforementioned issue dealing with the difficulty in replicating the real working conditions of adhesively bonded joints, several procedures were developed for taking into account, on one hand, the possibility of critical environmental conditions (in terms of moisture and temperature), on the other the occurrence of cyclically variable loads, which the most of the bonded joints are required to withstand. Focusing on the latter, the literature on adhesively bonded joints with polymeric substrates undergoing cyclic loading is not very wide, the only exception being the countless works dedicated to composite joints. Ashcroft et al. [9] found that an adhesively bonded composite double lap joint cyclically loaded can fail at a percentage of the static strength ranging from 26% to 62%. Harris and Fay [10] proved that the major percentage of the fatigue life was spent in initiating the crack at the edges. The stress concentration at the edges of the bonded area must be carefully taken into account, especially considering the effect providing on it by several geometric features of the bonded joints, such as the overlap length [11] and the adhesive thickness [12]. Nevertheless, the crack initiation stage is usually neglected by many authors, who chose to evaluate the fatigue life based only on the crack propagation phase. This often depended on the difficulty in precisely identifying the transition from the initiation to the propagation, whose measure strictly depends on the employed technique. In [13], the backface strain method was used to find that in the low load/high cycle range, the fatigue life in adhesively bonded composite joints was dominated by the crack initiation stage, which is also consistent with what occurs using metallic adherends [14]. Video microscopy was used in [15] to detect the crack onset and to find that the amount of fatigue life spent for the initiation was ranging from 20 to 70%, and it depended on materials, corner geometry, load level and presence of defects in the bondline. Most of the authors agree that the fatigue life of adhesively bonded joints is dominated by crack propagation. Fernandez et al. [16] used a fatigue crack growth approach to identify the evolution of strain energy release rate with the crack length in carbon/epoxy bonded joints. Huang et al. [17] applied the same method to GFRP/epoxy joints under cyclic mixed-mode load conditions, even using DIC to measure the deformation at the crack tip. Two alternative approaches are usually employed to analyze the fatigue response in bonded joints; namely, the stress-life and the fatigue-crack propagation approaches, respectively [18,19]. The benefits that surface pretreatments have over the static strength of adhesively bonded joints also extend to the fatigue response [20]. This is well-known for metallic adherends, where many treatments were successfully tested with respect to their capability to enhance the fatigue behavior of joints [21,22,23]. Among the authors, who spent some effort on joints with polymeric adherends, Kim et al. [24] used ϒ-glycidoxypropyltrimethoxysilane to create a thin interphase layer in composite/steel joints with the intent to increase their adhesion strength and the fatigue life under dynamic loading. Nevertheless, the literature over the fatigue behavior of joints realized with polymeric adherends without reinforcing fibers is still very limited. The aim of this work is to try to explore the feasibility of enhancing the fatigue response of polyamide adhesively bonded single lap joints (SLJ) by means of the application of APPT as a surface pretreatment. Two process parameters, namely the surface-to-nozzle distance and the treatment speed, were varied between two levels in order to investigate the influence that each of them has over the fatigue behavior. Moreover, the simple abrasion and the abrasion followed by APPT performed using only a combination of the process parameters among those considered were tested with the intent to assess the possible enhancement gained with respect to separately using abrasion or plasma treatment, respectively. The assessment was performed by using the stress–life approach and by evaluating the number of cycles to failure resulting from the application of a sinusoidal loading cycle. The ANOVA was applied to the stored data to give statistical strength to the argued conclusions. For a representative set of specimens, both the crack initiation and propagation stages were monitored by means of the digital image correlation (DIC) technique in order to assign to each stage the corresponding percentage of fatigue life.

## 2. Materials and Methods

### 2.1. Materials and Geometry

The experimental setup was arranged as described in a previous work [25], in which the APPT was performed over adherends realized with several polymers, including the one used in this work, with the intent to assess its effect over the quasi-static response of SLJ and to compare it with the one provided by other classic pretreatments (e.g., abrasion). In particular, polyamide was chosen for its applicability to different industrial fields (e.g., automotive, electronics, manufacture of bearing cages, pumps, pneumatic connectors and other components of industrial machinery and equipment) [26]. The properties of the employed polyamide, supplied by Ensinger (Milano, Italy), were collected in Table 1. The adhesive selected was Teroson PU 9225 (Henkel Italia, Milano, Italy), which is a commercial polyurethane bicomponent adhesive. As specified in the supplier technical data sheet [27], the adhesive curing time is approximately 5 h after mixing at room temperature.

**Figure 1:**
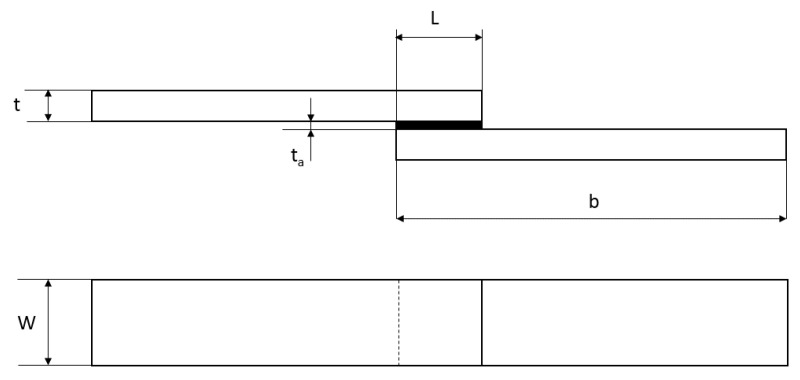
Single lap joint geometry.

For the mechanical tests, SLJ was chosen as specimens’ geometry, and ASTM D 3163 was assumed as a reference [28]. Figure 1 and Table 2 provide information regarding the main dimensions of the specimens.

### 2.2. Surface Pretreatments

Four pretreatments were applied over the surfaces aimed to be bonded together, namely:

(1) Degreasing: it was carried out by wiping the surfaces by means of a clean cloth soaked with Henkel 7063 cleaner (Henkel Italia, Milano, Italy);
(2) Abrasion: the pretreatment was performed using an aluminum Oxide, 320 grit sandpaper until the effect of the abrasion was barely visible. The abrasion was followed by wiping and degreasing by means of Henkel 7063 cleaner;
(3) APPT: the plasma treatment was performed by means of the Plasma Beam system, supplied by Diener (Diener electronic GmbH, Ebhausen, Germany) and equipped with a 300 W generator. The atmospheric air was used as both processes and cooling gasses. The treatment was carried out as described in [25]. The considered process parameters were the surface-to-nozzle distance (D) and the treatment speed (v). Every combination obtained by varying each parameter between two levels was tested: D could assume the value 5 mm or 10 mm, while v could be equal to 100 mm/s or 200 mm/s;
(4) Abrasion + APPT: even some specimens pretreated with both abrasion and plasma (with the only combination of parameters D = 5 mm and v = 100 mm/s) were realized in order to allow the assessment of the combined effect of the two treatments on the mechanical fatigue properties.

### 2.3. Surface Characterization

The morphologies of the surfaces, obtained after the surface pretreatment, were characterized with a Taylor Hobson TalySurf green light (Taylor Hobson Ltd, Leicester, UK) coherence correlation interferometry (CCI) noncontact automated optical profiler. For each pretreatment, 600 μm × 600 μm morphology maps were acquired with a resolution of 340 nm on the longitudinal plane and 1 nm on the vertical axis. The acquisitions were used to calculate the average surface roughness, Sa, according to ISO 25178-2:2012 [29].

### 2.4. Mechanical Characterization

The experimental test started from the characterization of the apparent quasi-static shear strength, evaluated as in Equation (1):

(1) $$\tau_{\mathrm{MAX}}=\frac{P_{\mathrm{MS}}}{\mathrm{WL}}$$

**Figure 2:**
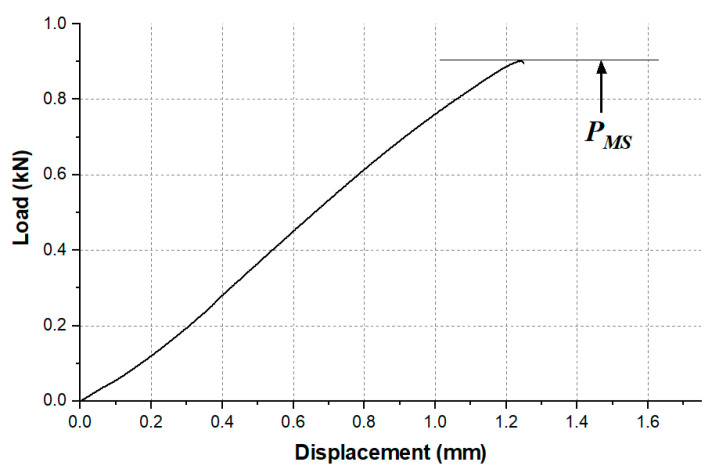
Example of load displacement curve of a quasi-static test.

The dimensions W and L were previously described in Figure 1, while PMS is the maximum load, as it is shown in the load–displacement curve provided as an example in Figure 2.

The static tests were executed in displacement control, at room temperature, in an Instron 440 electro-mechanical machine equipped with a 30 kN load cell (Instron, Torino, Italy). Following ASTM D 3163 [28], the test speed was set to 1.3 mm/min. Four samples for every surface treatment condition were tested. The test was carried out 1 week after the joint manufacturing, with the aim of ensuring that the polymerization was complete.

The fatigue tests were carried out at room temperature in an MTS 810 servo-hydraulic testing machine supplied by MTS Systems (Torino, IT) and equipped with a 5 kN load cell. ASTM D 3166 [30] was taken as a reference. The tests occurred in load control by applying a sinusoidal load wave characterized by a fixed frequency (f = 6 Hz) and load ratio (R = 0.1). The maxima loads applied were set in order to produce a number of cycles to failure included in the range between 1000 and 1,000,000.

The data resulting from the tests were analyzed using the stress–life approach, in particular the fatigue curve was plotted by evaluating the maximum component of the apparent shear stress, which took action during every cycle, τ_MAX_, and the number of cycles to failure, N, and by assuming a log-normal distribution of N according to Equation (2):

(2) $$N\cdot \tau_{\mathrm{MAX}}^{\mu }=k$$

where μ are the inverse slope and k^1/μ^ is the intercept of the corresponding curve plotted in a double logarithmic graph N-τ_MAX_.

**Figure 3:**
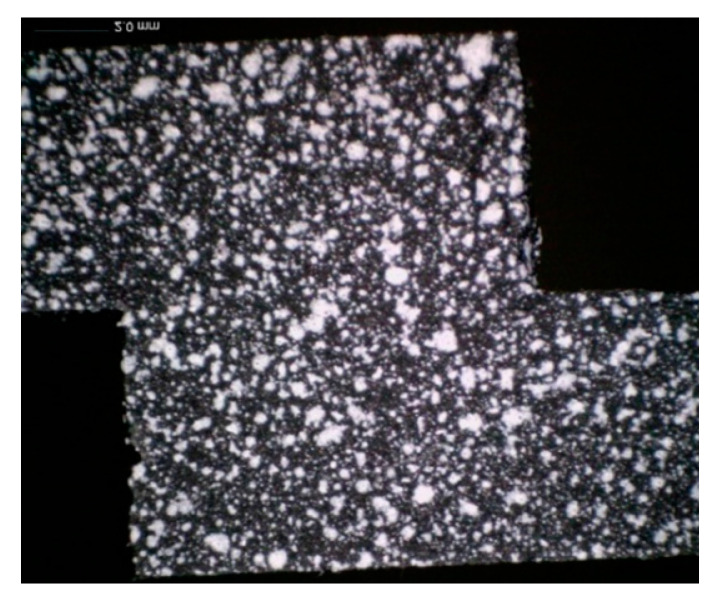
Example of the images of the side of the specimen used to perform the image correlation.

In order to understand the influence of the surface treatment on the development of the joint damage and failure, and, in particular, on the percentage of the fatigue life spent in crack onset and crack propagation, a set of representative specimens for every surface treatment was analyzed using the digital image correlation (DIC) technique. In particular, pictures of one side of the specimen (painted with appropriate speckles) were acquired at fixed time intervals along the fatigue test using a 5 MPx Dinolite digital camera (Almere, The Netherlands), as shown, for example, in Figure 3.

**Figure 4:**
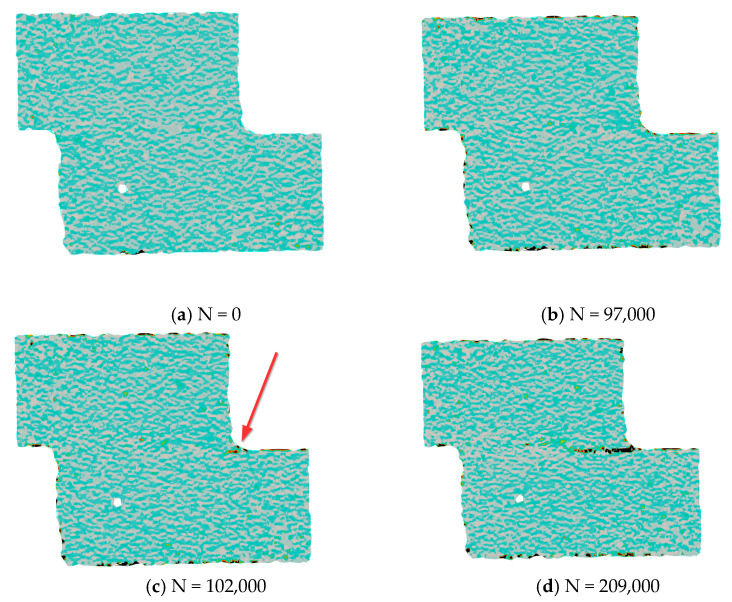

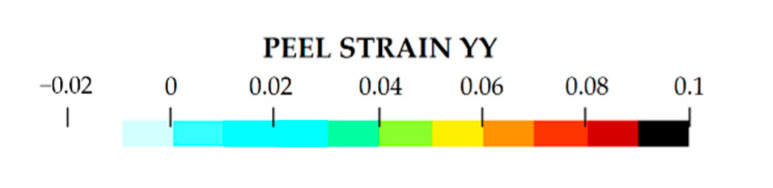
Example of peel strain values measured by digital image correlation during a test: (**a**) at the beginning of the test; (**b**) last acquisition before crack onset; (**c**) first acquisition after crack onset; (**d**) a few cycles before the final failure.

**Figure 5:**
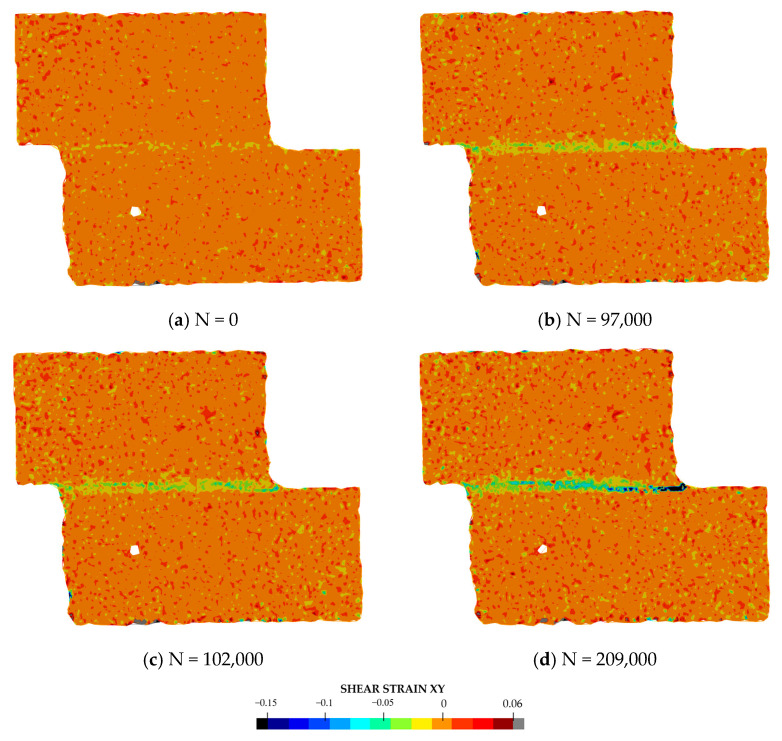
Example of shear strain values measured by digital image correlation during a test: (**a**) at the beginning of the test; (**b**) last acquisition before crack onset; (**c**) first acquisition after crack onset; (**d**) a few cycles before the final failure.

Later, the digital image correlation was performed with DICe open-source software [31], with the aim of recognizing the onset of the crack. An example of the results of the correlation of a fatigue test is given in Figure 4 (peel strain) and Figure 5 (shear strain). Four images are given: (a) at the beginning of the test, (b) last acquisition before the crack onset, (c) first acquisition after crack onset, and d) last acquisition before failure. It can be noticed that for a number of cycles equal to 209,000, just before the final failure (panel d), the presence of the defect is clearly noticeable for both the strain fields. Panels b and c of Figure 4 and Figure 5 show the two strain distributions at the photogram just before and after the onset of the defect. It can be noticed that the presence of a small defect is more evident when the peel strain is analyzed, while it is difficult to locate it by analyzing the shear strain. Therefore, the first strain field was used to identify the onset and the corresponding number of cycles. The crack was considered to be initiated when a small defect (0.1–0.2 mm) could be detected from the result of the correlation.

### 2.5. Statistical Analysis

In order to assess if the differences among series of data belonging to distinct families of specimens were significant, the results underwent statistical analysis. Specifically, an analysis of variance (ANOVA) was carried out with the intent to check if the data dispersion was such to produce a distribution of the results around the same average value, regardless of the applied pretreatment. In particular, the application of a two-factors and two-levels ANOVA design allowed to separately consider first the influence of the employed treatments and then the effect of every process parameter, limited to plasma treatments. Therefore, the two factors represented by the treatments (factor A = abrasion, factor B = APPT with D = 5 mm and v = 100 mm/s) and the two levels describing the occurrence of the specific treatment (level 1 = yes, level 2 = no) were able to allow the evaluation of the statistical weight covered by abrasion only, by plasma treatment only and by the combined use of them in determining any differences in the averages, while the second ANOVA took into account all and only the APPT joints, used D and v as factors and the employed values of the parameters as levels. The implementation of the ANOVA on the fatigue data was developed according to what was described in [32]. The steps of the procedure followed are detailed in Appendix A.

## 3. Results

### 3.1. Surface Characterization

**Figure 6:**
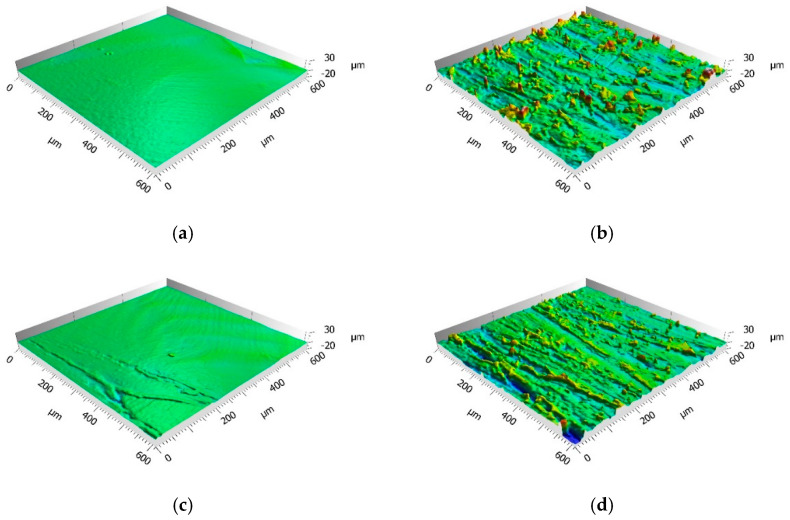
Example of the surface morphologies resulting from: (**a**) degreasing; (**b**) abrasion; (**c**) atmospheric pressure plasma treatment (APPT); D = 5 mm, v = 100 mm/s); (**d**) abrasion + APPT (D = 5 mm, v = 100 mm/s).

Figure 6 provides the morphology maps for degreased, abraded, plasma-treated and abraded + plasma-treated surfaces, while Table 3 gives the corresponding value of Sa.

It can be noticed that, as expected, the abrasion significantly increased the surface roughness, while on the opposite the plasma treatment did not alter the surface morphology, even if the plasma treatment was preceded by the abrasion (the only set of parameters D = 5 mm and v = 100 mm/s is shown since it represents the more aggressive treatment in terms of heat generated over the treated surface).

### 3.2. Static Tests Results

**Figure 7:**
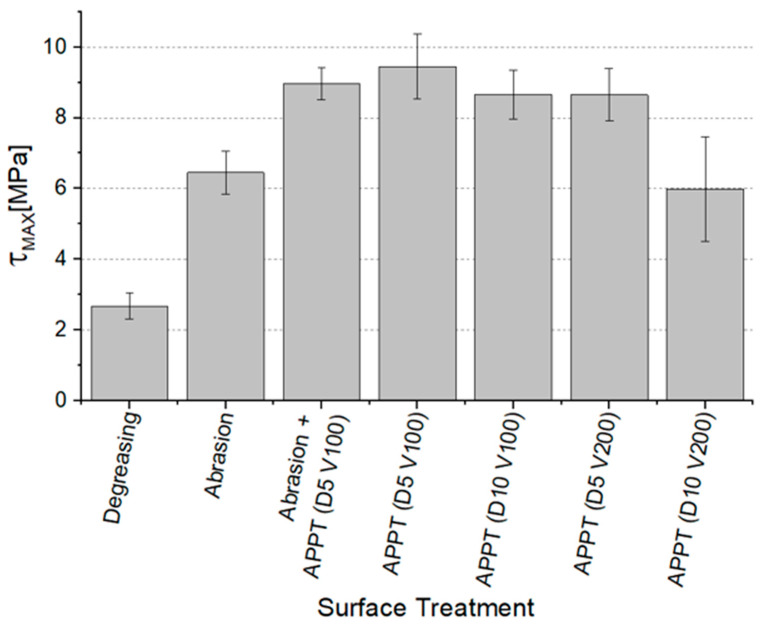
Average value and standard deviation of apparent shear strength for every set of specimens.

Figure 7 provides the average values and the standard deviation of the quasi-static apparent shear strength, τ_MAX_, for every set of specimens.

**Figure 8:**
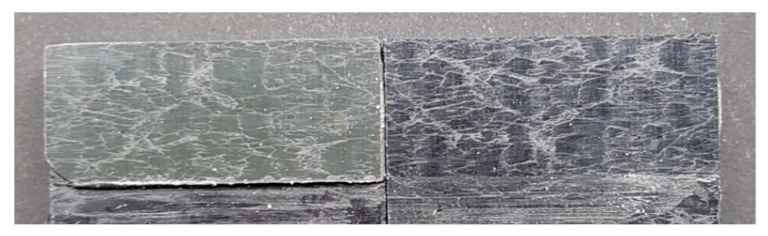
Fracture surface of the degreased specimen subjected to the quasi-static tensile test.

**Figure 9:**
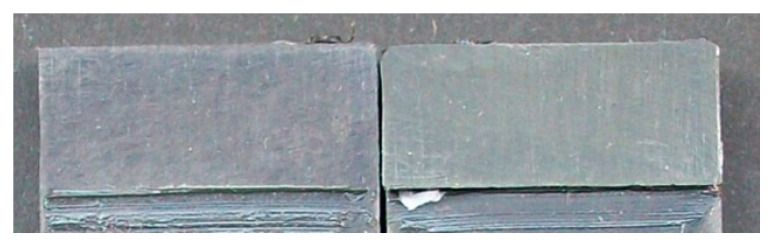
Fracture surface of the abraded specimen subjected to the quasi-static tensile test.

**Figure 10:**
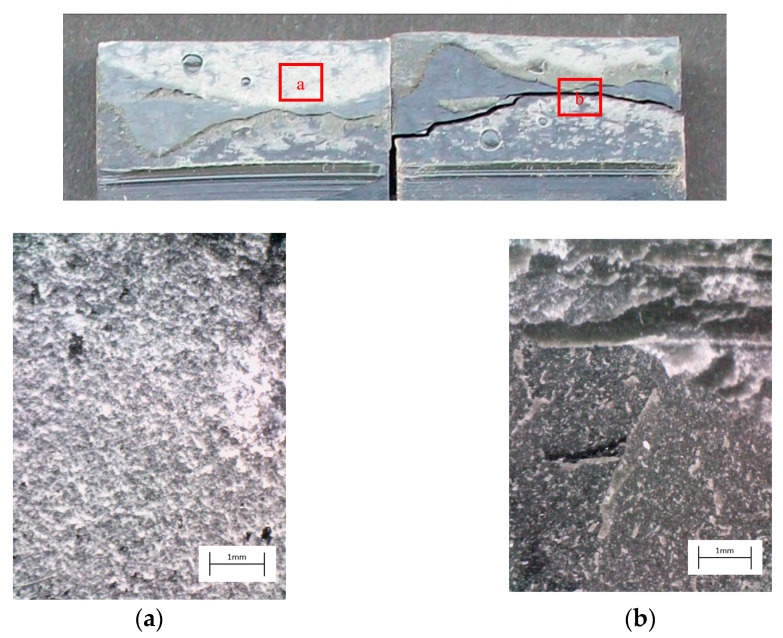
Fracture surface of a specimen pretreated with APPT with D = 5 mm and v = 100 mm/s and subjected to quasi-static tensile test, with magnified details (**a**) and (**b**) acquired by microscope.

**Figure 11:**
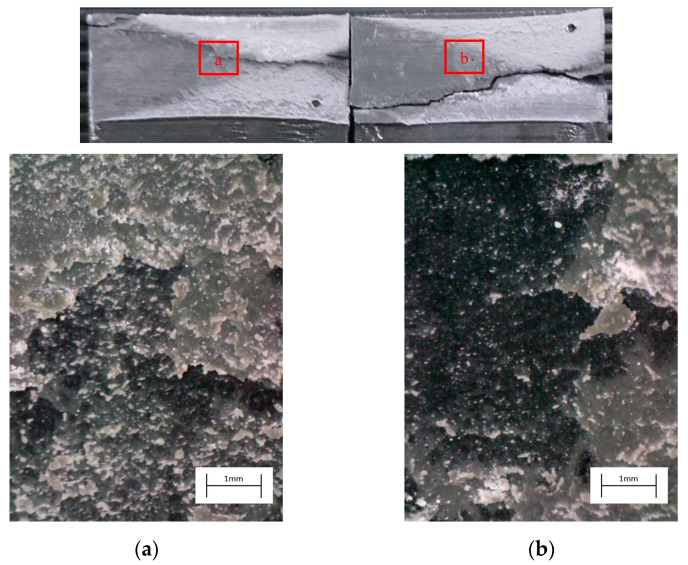
Fracture surface of a specimen pretreated with abrasion + APPT with D = 5 mm and v = 100 mm/s and subjected to quasi-static tensile test, with magnified details (**a**) and (**b**) acquired by microscope.

**Figure 12:**
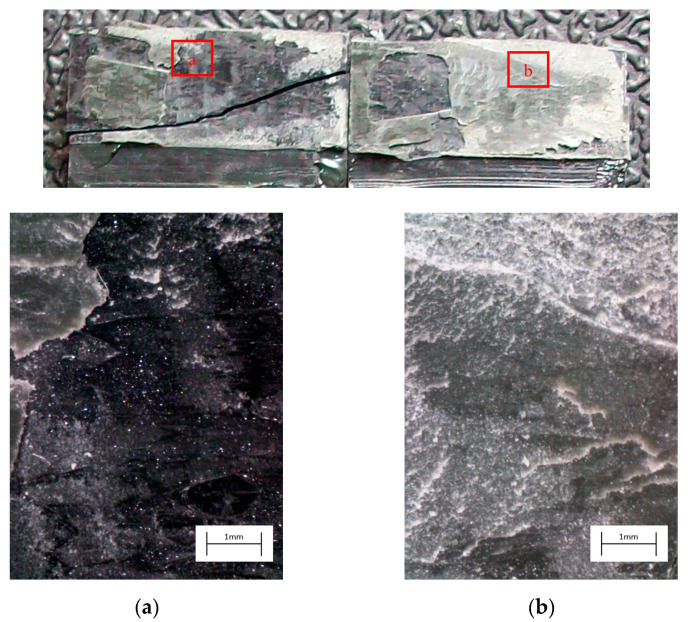
Fracture surface of a specimen pretreated with APPT with D = 10 mm and v = 100 mm/s and subjected to quasi-static tensile test, with magnified details (**a**) and (**b**) acquired by microscope.

**Figure 13:**
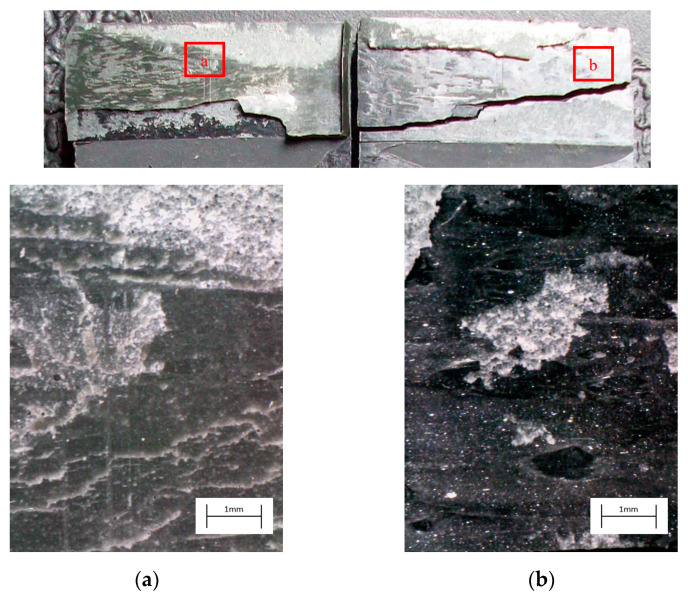
Fracture surface of a specimen pretreated with APPT with D = 5 mm and v = 200 mm/s and subjected to the quasi-static tensile test, with magnified details (**a**) and (**b**) acquired by microscope.

**Figure 14:**
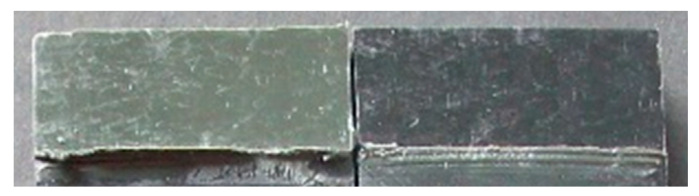
Fracture surface of a specimen pretreated with APPT with D = 10 mm and v = 200 mm/s and subjected to the quasi-static tensile test.

The degreased joints presented the lowest value of shear strength, and the associated fracture mode was always adhesive (Figure 8). The abrasion resulted in an increase of the mean apparent shear strength by approximately 140% with respect to the untreated value. Nevertheless, even the fracture surface of this class of specimens exhibited a completely interfacial failure, as it is possible to appreciate in Figure 9. The gain became further significant (about 261%) when the APPT with the lowest values of distance and speed was applied (Figure 10). It is worth noting the occurrence here of the characteristic stress-whitening associated with relevant energy dissipation mechanisms taking place during the crack propagation before the complete failure. Moreover, from details shown in Figure 10a,b) the presence of adhesive even in the areas where the crack seemed to propagate in a more interfacial way allowed us to assume that the fracture mechanism was mainly cohesive. The combination of abrasion and plasma treatment (performed with D = 5 mm and v = 100 mm/s) resulted in an average shear strength almost similar to the one of the respective plasma-treated joints, leading to the conclusion that chemical activation given by plasma treatment played a more important role than the surface roughening given by the abrasion. Furthermore, the fracture surfaces of abraded + APPT joints (Figure 11) did not significantly differ from those of the respective APPT treated joints. By considering the plasma treatment alone, increasing the distance from 5 to 10 mm (keeping fixed the test speed) or increasing the test speed from 100 mm/s to 200 mm/s (keeping untouched the distance) produced the same result in terms of both the average shear strength and the standard deviation, in particular resulting in a slight lowering of the strength by about 10% with respect to the maximum value. The fracture surfaces of these two sets, illustrated in Figure 12 and Figure 13, respectively, presented the same appearance, due to a failure, which could still be classifiable as mainly cohesive, but with a crack propagation occurring near to the interface and switching from a side to the other of the bonded zone. In both cases, indeed, there were some areas where the substrates were barely visible (as it is possible to appreciate in Figure 12a for the D10_v100 sample and Figure 13b for the D5_v200 case), but the higher amount of the surface of the detached adhesive resulted rough and winding, as one can see in Figure 12b and Figure 13a, where several layers of adhesive, through which the crack propagated, were apparent. Finally, a simultaneous increase of both the distance and the treatment speed determined a further decrease of the average shear strength to a value, which was close to the one belonging to the abraded samples and which was lower by 38% than the maximum value. The fracture surface consistently showed a completely adhesive failure, as in the abraded case (Figure 14). The results were consistent with the wettability trend detected in [25] with the static contact angle method in function of the same process parameters here considered. In particular, the surface free energy of the samples treated with D = 5 mm moved from slightly higher to slightly lower than 70 mN/m as the test speed v increased from 100 mm/s to 200 mm/s, while a treatment with D = 10 mm and v = 100 mm/s produced a surface energy approximately equal to 66 mN/m and finally treating with D = 10 mm and v = 200 mm/s resulted in a surface free energy around to 60 mN/m.

**Figure 15:**
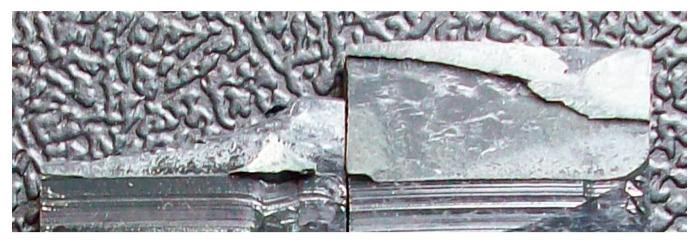
Fracture surface of a specimen pretreated with plasma using D = 5 mm and v = 100 mm/s.

It is worth noting that most of the specimens that showed the highest average shear strengths presented a mixed-mode of failure, as is shown in Figure 15 for a joint belonging to the D = 5 mm and v = 100 mm/s set. In particular, a mainly cohesive failure occurred within the bonded area, with the crack moving from one side to another, but the energy release and the bending resulting from the single lap configuration seemed to be such as to lead even to the breakage of the adherend.

**Figure 16:**
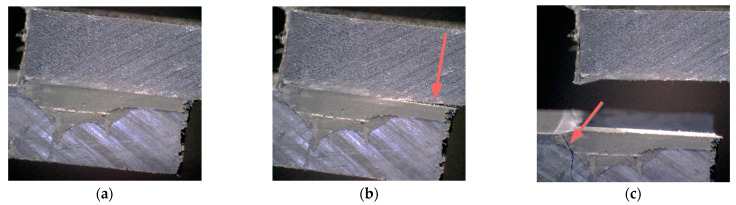
Example of development of substrate failure shown by some specimens: (**a**) undamaged specimen; (**b**) onset and propagation of failure within the adhesive layer (**c**) final failure with substrate cracking.

The development of this behavior was caught by observing the side of the specimen during a quasi-static test. Figure 16 shows three different phases of the joint failure and, in particular, the onset and propagation of the failure within the adhesive layer (b), followed by the failure of the substrate (c).

### 3.3. Fatigue Tests Results

**Figure 17:**
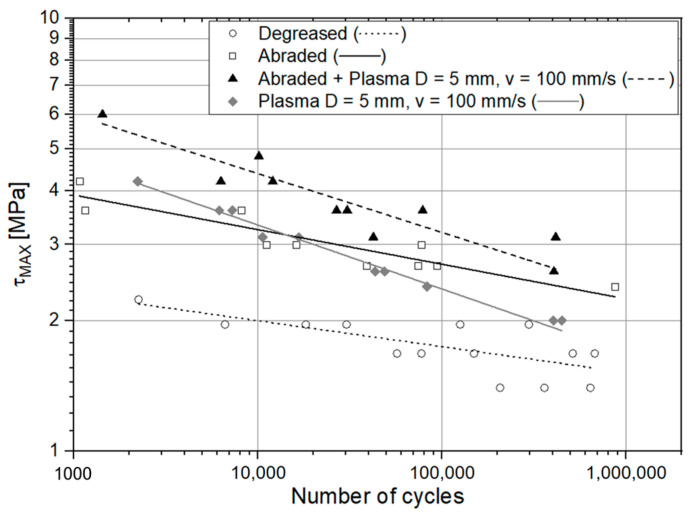
S-N curves for the following set of specimens: degreased, abraded, abraded + APPT (D = 5 mm and v = 100 mm/s) and APPT with D = 5 mm and v = 100 mm/s (the lines represent the power-law trend curves).

**Figure 18:**
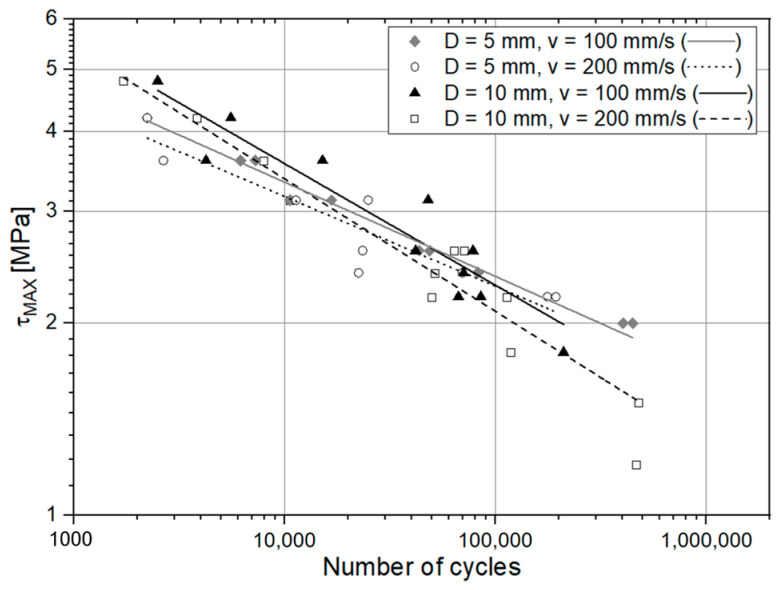
S-N curves for the specimens pretreated with APPT (the lines represent the power-law trend curves).

The S-N (Stress – Number of Cycles to failure) fatigue curves are plotted in two different figures for the sake of clarity. In Figure 17, a double logarithmic graph reporting the fatigue curves related to the specimens treated with degreasing, abrasion alone, abrasion + APPT with D = 5 mm and v = 100 mm/s and finally APPT (with the same combination of parameters) alone is provided. Figure 18 instead compares the fatigue response of the plasma-treated joints, allowing to discern the different effects provided by the tested configurations. Table 4 reports the values of the inverse slope μ, the intercept k^1/μ,^ and the coefficient of determination R^2^ evaluated for every curve.

**Figure 19:**
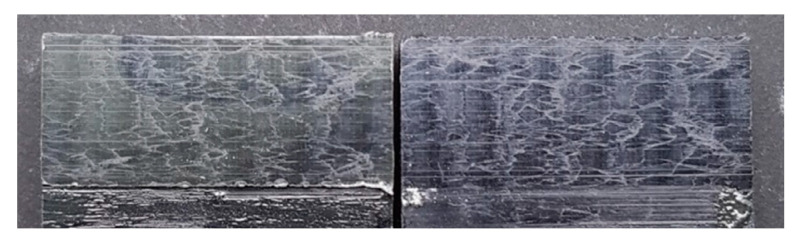
Fracture surface of the degreased specimen subjected to fatigue test.

**Figure 20:**
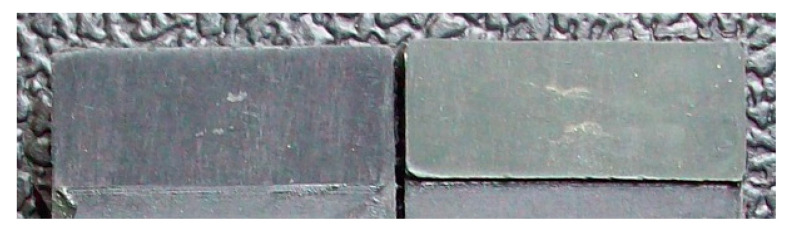
Fracture surface of the abraded specimen subjected to fatigue test.

**Figure 21:**
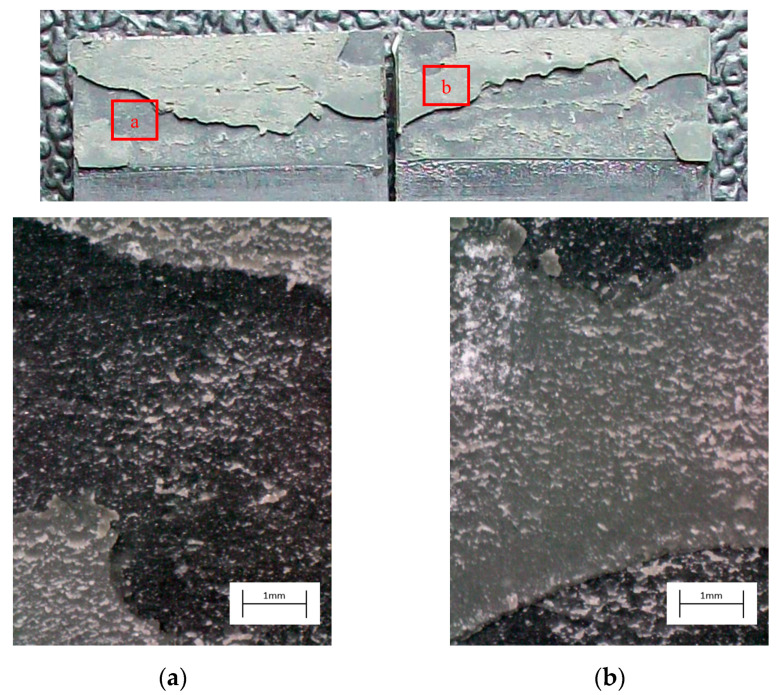
Fracture surface of a specimen pretreated with abrasion + APPT with D = 5 mm and v = 100 mm/s and subjected to fatigue test, with magnified details (**a**) and (**b**) acquired by microscope.

**Figure 22:**
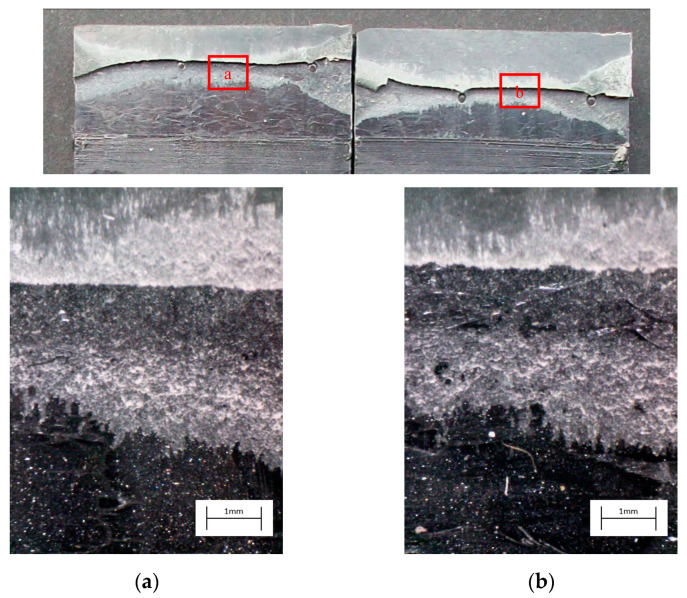
Fracture surface of a specimen pretreated with APPT with D = 5 mm and v = 100 mm/s and subjected to fatigue test, with magnified details (**a**) and (**b**) acquired by microscope.

**Figure 23:**
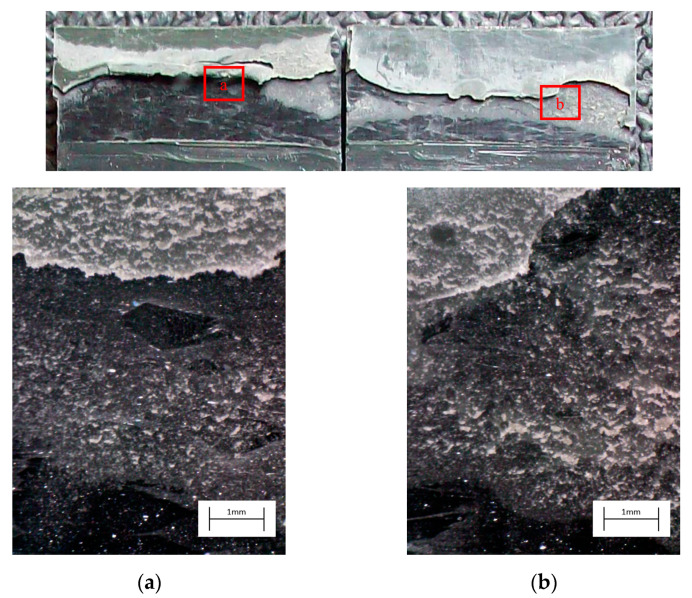
Fracture surface of a specimen pretreated with APPT with D = 10 mm and v = 100 mm/s and subjected to fatigue test, with magnified details (**a**) and (**b**) acquired by microscope.

**Figure 24:**
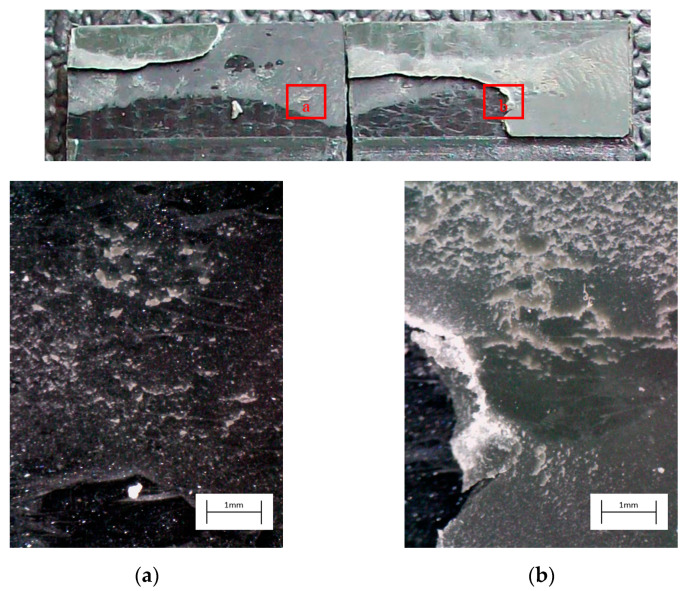
Fracture surface of a specimen pretreated with APPT with D = 5 mm and v = 200 mm/s and subjected to fatigue test, with magnified details (**a**) and (**b**) acquired by microscope.

**Figure 25:**
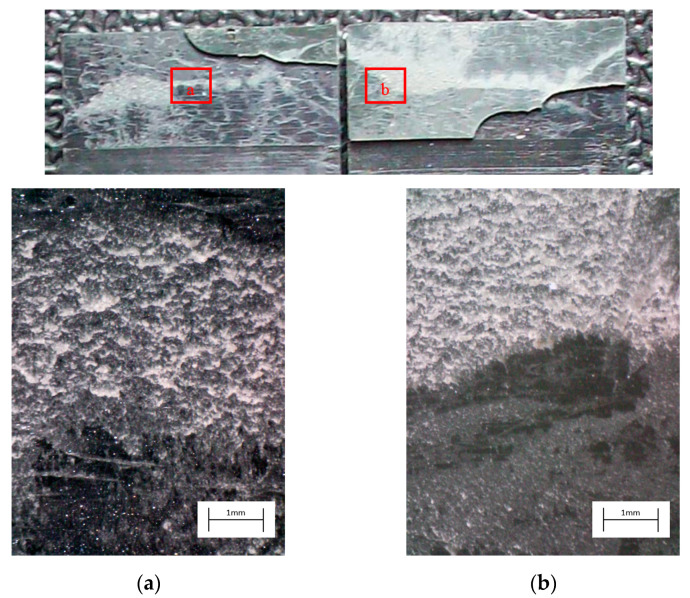
Fracture surface of a specimen pretreated with APPT with D = 10 mm and v = 200 mm/s and subjected to fatigue test, with magnified details (**a**) and (**b**) acquired by microscope.

The curve associated with the simply degreased samples, whose static shear strength was the lowest among the tested ones, was characterized by the lowest slope and the lowest intercept, confirming the poor performance also in the case of fatigue loading. The low coefficient of determination of this group of specimens confirms the high data dispersion, which appears from the graph in Figure 17. The fracture surface of these specimens resulted always smooth and characterized by a completely adhesive failure, as can be seen in Figure 19. The same comments are substantially replicable even for the abraded specimen family, characterized by values of slope and intercept slightly higher than those of the degreased samples curve, but sufficient to place the whole curve on top of the degreased one. However, the fracture surface showed in Figure 20 appeared analogous to the one of the degreased specimens. Indeed, the family of the abraded + APPT joints was apparently the one exhibiting the best fatigue performance, both at low and at high number of cycles to failure. The combination of the two treatments appeared, therefore, as the best opportunity, among the considered ones. In the samples belonging to this set, the crack propagated near to the interface and with at least one switch of side, but the failure resulted predominantly cohesive, as it was revealed by analyzing the magnified details provided in Figure 21a,b), where some traces of adhesive were detected even on the portions of the fracture surface closest to the interface. By performing the APPT alone using the same parameters already employed in combination with abrasion, it was possible to achieve a good performance at a low number of cycles, while the fatigue response worsened at a high number of cycles, making the failure occur at low shear stress values with respect even to the abraded family. Moving to compare to each other the trends exhibited by the purely plasma-treated samples and shown in Figure 18, it is worth noting that all the curves appear gathered, and the differences between a set and another are small, especially considering the significant scatter shown by the tests. However, by considering the fitted power-laws, it is possible to identify a trend related to the value of surface-to-nozzle distance: the curves related to the specimens treated with D = 5 mm presented the lowest slope if compared with those treated with D = 10 mm. An observation of the fracture surfaces (Figure 22, Figure 23, Figure 24 and Figure 25) revealed how, for all the purely APPT joints, the failure occurred in a mixed-mode, since the magnified details acquired with a microscope revealed how on the surface each one of the three phases represented by a thick layer of adhesive, thin layer of adhesive and bare substrate, respectively, was present, which meant that the crack moved at most once from a side to another. Nevertheless, the adhesive surface appearance resulted less smooth than the one detectable on the abraded specimen and, unlike the case of the abraded joints, the stress-whitening phenomenon occurred.

The results of the ANOVA performed with the intent to assess the influence of the treatments are provided in Table 5 and Table 6, where the evaluated values of the F-distribution and *p*-value were also listed.

The results of the analysis clearly show how the occurrence of the single treatments has a significant effect on the fatigue behavior of the joints. In particular, the probability that the abrasion does not produce a family with a statistically different average fatigue strength with respect to the class of not abraded specimens is lower than 0.00001, while the same quantity evaluated for plasma treatment is equal to 1.7%. Indeed, the employment of both treatments does not seem to produce a statistically significant effect, which means that there is no interaction between the treatments. Therefore, the effects of abrasion and of plasma treatment seem to act quite independently from each other, which confirms the prevalence of the AB + PL data with respect to the other set resulting from the analysis of the fatigue curves.

Considering the outcomes from ANOVA applied to the assessment of the influence of the APPT plasma parameters, the results were in general rather scattered; however, some assertion can be drawn by considering the p-values. Their values are quite high, indicating that there is not the predominant effect of one of the factors or of the interaction on the fatigue life. However, the p-value associated with the surface-to-nozzle distance effect is the lowest, and thus, this factor appears to be the most significant. This is consistent with the trend identified looking at the S-N curves of the plasma-treated joints, from which an increase of the fatigue behavior at a high number of cycles by means of a decrease of the parameter D was noticed. Indeed, the p-values related to the treatment speed and the interaction between the two considered parameters result very high, leading to conclude that differentiation of the treatment speed or a simultaneous change of both the parameters among the values employed in this work does not produce a significant differentiation of the fatigue response.

The analysis of initiation and propagation of the defect carried out using the DIC technique was performed for at least four specimens for each surface pretreatment, considering specimens failed within a range of 50,000–500,000 cycles. Table 7 shows the result of the analyses, and it can be noticed that the degreased joints showed a percentage of life spent in the initiation of the defect considerably higher than the other pretreatments. This means that once the defect was created, the propagation of the defect at the interface between the adhesive and the substrate rapidly led to the failure of the specimen.

On the opposite, the combination of abrasion and plasma treatment seems to lead to a higher percentage of fatigue life spent in propagation, justifying the higher fatigue life of specimens that underwent this surface pretreatment.

## 4. Conclusions

In this work, a characterization of the fatigue behavior of adhesively bonded single lap joints realized with polyamide substrates and a polyurethane adhesive was described. The aim of the work was to identify the effects on the fatigue durability of the joints provided by performing different treatments on the surface of the substrates before bonding. The investigated pretreatments were degreasing, abrasion and APPT with different combinations of surface-to-nozzle and treatment speed. Abrasion and APPT were also combined with each other to verify the possible improvement conferred to the mechanical response of the joints. Several quasi-static tests in order to investigate the differences in the apparent shear strength between different sets of specimens were followed by the execution of a series of cyclically loaded tests. The stress–life approach was chosen to assess the variation of the number of cycles to failure according to the applied pretreatment or, in the APPT case, to the adopted process configuration. The DIC method was also employed to try to segregate the portion of life spent to initiate the defect from the percentage of a life dedicated to the crack propagation.

The results of the quasi-static tests showed that the apparent shear strength increased from the degreased to the abraded and finally to the plasma-treated samples. In particular, among this last group, a progressive decrease of the strength was recorded by progressively rising up the surface-to-nozzle and the treatment speed values employed for the treatment. When abrasion was simultaneously used with plasma, the response was equal to the one of the respective plasma group. The associated failure modes detectable from the appearance of the fracture surfaces were consistent with the strength results. The measure of the surface roughness of samples treated with different methods allowed us to conclude that the influence on the mechanical behavior of the chemical changes induced by the plasma treatment on the surface transcends the benefits conferred by the morphological modifications provided by abrasion.

The S-N curves revealed a similar trend to the static tests, with some exceptions. Even in this case, the degreased sample response appeared to be the worst among the considered groups, while the combination of abrasion and plasma treatment assured the highest fatigue strength, both at the high and a low number of cycles to failure in the investigated range. Between these two extremes, the whole data of the plasma-treated joints were gathered. A hierarchy between them could, however, be established by noticing that an increase of the surface-to-nozzle value led to an increase of the fatigue strength at a high number of cycles to failure. Even in this case, the analysis of the curve was accompanied by an assessment of the fracture surfaces. The DIC acquisition and data elaboration allowed to relate the highest fatigue strength of abraded + APPT specimens with their higher percentage of life spent in the propagation phase, compared to the joints made with other pretreatments. The application of the ANOVA allowed conferring statistical strength to the comments resulting from the analysis of data.

## Figures and Tables

**Table 1:** Details of the polymeric material used for the joint production.

Material	Polyamide (PA)
Supplier	Ensinger
Commercial name	Tecamid 66 MO (PA66)
Color	Black
Modulus of elasticity (MPa)	3200
Yield stress (MPa)	83
Additives	< 2 w/w% (black pigment and molybdenum bisulfite)

**Table 2:** Single lap joint dimensions.

Dimension	Value in mm
Adherend thickness, t	6.6
Adhesive thickness, ta	0.3
Overlap length, L	10
Adherend length, b	100
Adherend width, W	25

**Table 3:** Average surface roughness, Sa, for the considered pretreatments.

Surface Treatment	Sa (μm)
Degreasing	0.45
Abrasion	3.15
APPT (D = 5 mm, v = 100 mm/s)	0.49
Abrasion + APPT (D = 5 mm, v = 100 mm/s)	2.85

**Table 4:** Regression coefficients of every S-N fatigue curve corresponding to a set of specimens.

Surface Treatment	µ (1/ln(MPa))	k^1/µ^ (MPa)	R^2^ (-)
Degreasing	15.15	3.66	0.54
Abrasion	12.82	6.62	0.83
Abrasion + APPT (D = 5 mm, v = 100 mm/s)	8.47	12.87	0.82
APPT (D = 5 mm, v = 100 mm/s)	6.80	13.00	0.99
APPT (D = 10 mm, v = 100 mm/s	5.00	22.45	0.89
APPT (D = 5 mm, v = 200 mm/s)	7.52	10.69	0.86
APPT (D = 10 mm, v = 200 mm/s)	4.52	26.03	0.94

**Table 5:** Results of ANOVA applied to a 2-by-2 experiment (A1/B1 = AB_D5_v100, A1/B2 = AB, A2/B1 = D5_v100, A2/B2 = UN).

Surface Treatment	Sum of Squares	DOF	Variance	Fisher’s Ratio	*p*-Value
Effect of abrasion	0.03029	1	0.03029	24.430	<0.00001
Effect of plasma treatment	0.00750	1	0.00750	6.052	0.017
Interaction	0.00037	1	0.00037	0.297	0.588
Error	0.05951	48	0.00124	-	-

**Table 6:** Results of ANOVA applied to a 2-by-2 experiment (A1/B1 = D5_v100, A1/B2 = D10_v100, A2/B1 = D5_v200, A2/B2 = D10_v200).

Surface Treatment	Sum of Squares	DOF	Variance	Fisher’s Ratio	*p*-Value
Effect of distance, D	0.00741	1	0.00740	5.506	0.024
Effect of treatment speed, v	0.00090	1	0.00090	0.336	0.418
Interaction	0.00057	1	0.00057	0.427	0.517
Error	0.05379	40	0.00134	-	-

**Table 7:** Percentage of fatigue life spent for initiation of defect for the considered pretreatments.

Surface Treatment	Percentage of Fatigue Life Spent for Initiation of Defect
Degreasing	70 ± 7%
Abrasion	56 ± 7%
APPT (D = 5 mm, v = 100 mm/s)	61 ± 5%
Abrasion + APPT (D = 5 mm, v = 100 mm/s)	50 ± 6%

## Data Availability

Data available on request. The data presented in this study are available on request from the corresponding author.

## Appendix A

The generic design of experiments for each of the two performed ANOVA is illustrated in Table A1.

**Table A1:** Generic design of experiments for ANOVA.

Factor B	Factor A–Level A1	Factor A–Level A2	Mean Values (Row)
**Level B1**	T11	T21	S1.
**Level B2**	T12	T22	S2.
**Mean values (column)**	S.1	S.2	S..

For each analysis, the quantities Tij were evaluated as the 10-base logarithm of τ_MAXij_, where the subscripts “i” and “j” refers to the set of specimens placed at the intersection of the i-th row and the j-th column. The row and column mean values were determined as in Equations from (A1) to (A4):

(A1) $$S_{1.\;}=\frac{T11+T21}{2}$$

(A2) $$S_{2.\;}=\frac{T12+T22}{2}$$

(A3) $$S_{.1\;}=\frac{T11+T12}{2}$$

(A4) $$S_{.2\;}=\frac{T21+T22}{2}$$

The overall mean value was then evaluated as in Equation (A5).

(A5) $$S_{..\;}=\frac{T11+T21+T12+T22}{4}$$

Considering that the row and column mean values were averaged between two functions, the sum of squares between rows (SSBR) and the sum of squares between columns (SSBC) were evaluated through the computation of the integral means over the life range between N_A_ = 10^3^ and N_B_ = 10^6^ cycles, as shown in Equations (A6) and (A7), respectively, and by using in both cases a weight factor equal to 2:

(A6) $$\mathrm{SSBR}=\;2\cdot \frac{1}{N_{B}-N_{A}}\cdot \int_{N_{A}}^{N_{B}}\left[{\left(S_{1.}-S_{..}\right)}^{2}+{\left(S_{2.}-S_{..}\right)}^{2}\right]\cdot \mathrm{dN}$$

(A7) $$\mathrm{SSBC}=\;2\cdot \frac{1}{N_{B}-N_{A}}\cdot \int_{N_{A}}^{N_{B}}\left[{\left(S_{.1}-S_{..}\right)}^{2}+{\left(S_{.2}-S_{..}\right)}^{2}\right]\cdot \mathrm{dN}$$

The interaction sum of squares (SSI) and the sum of squares for error (SSE) were instead evaluated as in Equations (A8) and (A9), respectively:

(A8) $$\begin{array}{cc} \mathrm{SSI} & =\frac{1}{N_{B}-N_{A}}\cdot \int_{N_{A}}^{N_{B}}[{\left(T11-S_{1.}-S_{.1}+S_{..}\right)}^{2}+{\left(T21-S_{1.}-S_{.2}+S_{..}\right)}^{2}\\ & \;\;\;+{\left(T12-S_{2.}-S_{.1}+S_{..}\right)}^{2}+{\left(T22-S_{2.}-S_{.2}+S_{..}\right)}^{2}]\cdot \mathrm{dN} \end{array}$$

(A9) $$\mathrm{SSE}=\;\overset{n}{\underset{i=1}{\sum }}\overset{p}{\underset{j=1}{\sum }}\overset{r}{\underset{k=1}{\sum }}{\left(\tau_{\mathrm{ijk}}-T_{\mathrm{ij}}\right)}^{2}$$

where τ_ijk_ is the k-th measured average shear strength belonging to the set placed at the intersection between the i-th row and the j-th column.

The Fisher’s ratios aimed to assess the influence of factors A or B, merely, or of their interaction, were evaluated as the ratio between SSBC, SSBR or SSI, respectively, and SSE, providing that every sum of square had been previously divided for the number of the related degrees of freedom.
